# Inhibition of endothelial cell functions and of angiogenesis by the metastasis inhibitor NAMI-A

**DOI:** 10.1038/sj.bjc.6600176

**Published:** 2002-03-18

**Authors:** A Vacca, M Bruno, A Boccarelli, M Coluccia, D Ribatti, A Bergamo, S Garbisa, L Sartor, G Sava

**Affiliations:** Department of Biomedical Sciences and Human Oncology, University of Bari, Policlinico, Piazza Giulio Cesare 11, I-70124 Bari, Italy; Department of Human Anatomy and Histology, University of Bari, Policlinico, Piazza Giulio Cesare 11, I-70124 Bari, Italy; Fnd. Callerio, Via A. Fleming 22-31, 34127, Trieste, Italy; Institute of Histology and Embryology, University of Padova, via G. Colombo 3 35122 Padova, Italy; Department of Biomedical Sciences, University of Trieste, via L Giorfiere 7 34127, Trieste, Italy

**Keywords:** angiogenesis, chemotaxis, endothelial cell, MMPs, ruthenium

## Abstract

NAMI-A is a ruthenium-based compound with selective anti-metastasis activity in experimental models of solid tumours. We studied whether this activity was dependent on anti-angiogenic ability of NAMI-A. We thus investigated its *in vitro* effects on endothelial cell functions necessary for angiogenesis to develop, as well as its *in vivo* effects in the chick embryo chorioallantoic membrane model. Endothelial cell proliferation, chemotaxis, and secretion of the matrix-degrading enzyme metalloproteinase-2 were inhibited by NAMI-A in a dose-dependent manner, and without morphologic signs of cell apoptosis or necrosis. Lastly, NAMI-A displayed a dose-dependent *in vivo* anti-angiogenic activity in the chorioallantoic membrane model. These data suggest that the anti-angiogenic activity of NAMI-A can contribute to its anti-metastatic efficacy in mice bearing malignant solid tumours.

*British Journal of Cancer* (2002) **86**, 993–998. DOI: 10.1038/sj/bjc/6600176 www.bjcancer.com

© 2002 Cancer Research UK

## 

Ruthenium-based compounds have recently received an increased attention as tools for investigating new anti-tumour agents (Clarke and Stubbs, 1996; Sava *et al*, 1999a). Some of them have reached an advanced milestone in the pre-clinical investigation (Keppler *et al*, 1990; Sava *et al*, 1998a) and one, namely imidazolium *trans*-imidazoledimethylsulfoxidetetrachlororuthenate (NAMI-A) (Mestroni *et al*, 1998), is successfully completing a phase I clinical trial at the Netherlands Cancer Institute of Amsterdam (J Schellens, personal communication). The initial evidences on the mode of action of NAMI-A, although far from the clear elucidation, showed this compound free of direct cytotoxicity for tumour cells and of effects on primary tumour growth (Sava *et al*, 1998a; Bergamo *et al*, 1999; Zorzet *et al*, 2000). Accumulated evidences indicated that NAMI-A inhibits metastasis growth by a selective effect on the relationship between metastatic cell and host environment, the importance of which for cell survival is well documented (Gregoire and Lieubeau, 1995; Nicolson and Menter, 1995; Radinsky, 1995). NAMI-A stimulated fibrosis growth at primary tumour site, with increased thickness of tumour capsule, cohesion among tumour cells and reduced vascular invasion of tumor mass (Sava *et al*, 1998a). Importantly, NAMI-A differs from most of the so-called anti-metastatic agents in that it is not only active in preventing metastasis formation but shows also good activity in inhibiting those already formed and in advanced stage of growth, this effect being very likely responsible for the increased survival time of the treated animals (Sava *et al*, 1999b). Since the metastasis occurrence is angiogenesis-dependent (Carmeliet and Jain, 2000) we assessed the hypothesis that the anti-metastasis activity of NAMI-A was correlated, at least partly, with its anti-angiogenic properties.

## MATERIALS AND METHODS

### Compound and treatment

Imidazolium *trans*-imidazoledimethylsulfoxide tetrachlororuthenate, ImH[*trans*-RuCl_4_(DMSO)Im] (NAMI-A), was prepared according to already reported procedures (Mestroni *et al*, 1998), and diluted stepwise from 240 μM to 5 μM with the culture medium of endothelial cells.

### Cell cultures

The human umbilical vein endothelial cells (HUVEC) were prepared as described previously (Bussolino *et al*, 1992) and grown in Petri dishes coated with 1% gelatin (Sigma Chemical Co, St Louis, MO, USA) in M199 medium supplemented with 20% foetal calf serum (FCS), 0.02% extract of bovine brain, and 0.01% porcine heparin (both from Sigma). The human endothelial-like immortalised cell line EA.hy926, derived from the fusion of HUVEC with the lung carcinoma cell line A549 (Edgell *et al*, 1983) was maintained in Dulbecco's modified Eagle's medium (DMEM) supplemented with 10% heat-inactivated FCS, 1% glutamine, amphotericin B (2.5 mg ml^−1^), penicillin (100 U ml^−1^), and streptomycin (50 mg ml^−1^). The neuroblastoma SK-N-BE and fibrosarcoma HT-1080 human cell lines were cultured in DMEM supplemented with 10% FCS, 2 mM glutamine and penicillin/streptomycin, 100 U ml^−1^ and 50 mg ml^−1^ respectively; NIH3T3 mouse embryo fibroblasts (American Type Culture Collection, ATCC, Rockville, MD, USA) were cultured in DMEM supplemented with 10% FCS and 1% glutamine.

### Preparation of conditioned media

The conditioned media (CM) of HUVEC, EA.hy 926, SK-N-BE, HT-1080 and NIH3T3 cells were prepared by incubating sub-confluent cells in a T25 flask with 6 ml of serum-free medium (SFM) for 24 h. The supernatant was collected under sterile conditions, centrifuged sequentially at 1200 and 12 000 rpm for 10 min to eliminate debris, and stored at −20°C.

### Proliferation assay

HUVEC or EA.hy926 endothelial cells were plated in 96-well plates (2.5×10^3^ cells well^−1^) in complete medium. After 24 h from seeding (day 0) and on days 2 and 4, the medium was replaced (in quadruplicate) with complete medium containing NAMI-A, with complete medium alone (positive control), or with starvation SFM (negative control). Cell number was estimated on day 6 by the colorimetric method of Kueng *et al* (1989). Briefly, cells were fixed for 15 min at room temperature with 2.5% glutaraldehyde, stained for 20 min with 0.1% crystal violet in 20% methanol, solubilised with 10% acetate, and read at 595 nm (Microplate Reader 3550, Bio-Rad Laboratories, Richmond, CA, USA). Cell number was derived from a calibration curve set-up with a known number of cells. NAMI-A effects upon endothelial cell proliferation were evaluated also after treatment times shorter than 144 h (48 h, 72 h and 96 h), as well as in the same experimental conditions used for chemotaxis and matrix metalloproteinase assays, i.e., after 24- and 18-h treatment times.

### Chemotaxis assay

This was carried out by the Boyden chambers as described previously (Vacca *et al*, 1999). HUVEC or EA.hy926 endothelial cells, pre-treated for 24 h with NAMI-A, were treated with trypsine (0.05%) and acetate (0.02%), collected by centrifugation, re-suspended in DMEM supplemented with 0.1% bovine serum albumin (BSA, Sigma Chemical Co), and seeded in triplicate in the upper compartment of a Boyden chamber (1.2×10^5^ cells/400 μl). The lower compartment was filled with 200 μl of the NIH3T3 CM as chemo-attractant or with DMEM supplemented with 0.1% BSA as negative control (to evaluate random migration). The two compartments were separated by a polycarbonate filter (12 μm pore size, Nucleopore, Costar, Cambridge, MA, USA) coated with 0.005% gelatine to allow cell adhesion. After 6-h incubation in humidified 5% CO_2_ air at 37°C, cells on the upper side of the filter were removed, whereas those which had migrated to the lower side were fixed in absolute ethanol, stained with toluidine blue, and counted in 5 to 8 oil immersion fields at 400×.

### MMP-2 sodium dodecylsulfate-polyacrylamide gel electrophoresis (SDS–PAGE) activity

#### Effect on MMP-2 secretion by EA.hy926 cells

T25 flasks of EA.hy926 cells at 80% confluence were rinsed twice with serum-free DMEM and incubated for 18 h in this medium either alone (positive control) or in the presence of NAMI-A. Culture media were collected, and the total protein content measured by the Bradford method (Bio-Rad Laboratories), using BSA as standard. To visualise the gelatinolytic activity of MMP-2 secreted in the CM, 20 μg aliquots of CM proteins were applied to 7.5% SDS–PAGE co-polymerised with type A gelatine from porcine skin (Sigma Chemical Co) at a final concentration of 0.6 g l^−1^. After electrophoresis in dual laboratory system (Protean II, Bio-Rad Laboratories), gels were washed in 2.5% Triton for 1 h to remove SDS, incubated for 18 h at 37°C, and then stained with 0.1% Coomassie brilliant blue. The gelatinolytic regions were observed as white bands against a blue background. MMP activity was measured by scoring the intensity of bands by computerised image analysis (APPLE, Computer Inc, Cupertino, CA, USA).

### Chick embryo chorioallantoic membrane (CAM) assay and quantification of the angiogenic response

Fertilized White Leghorn chicken eggs (20/group) were incubated under constant humidity at 37°C. On the third day of incubation, a square window was opened in the shell after removal of 2–3 ml of albumen so as to detach the developing CAM from the shell. The window was sealed with a glass of the same dimension, and the eggs were returned to the incubator. On day 8, 1 mm^3^ sterilized gelatin sponges (Gelfoam, Upjohn Co, Kalamazoo, MI, USA) loaded with 3 μl of PBS (negative control), or containing 1.0 μg per sponge of recombinant fibroblast growth factor-2 (FGF-2, R&D System, Abingdon, UK) alone (positive control) or together with NAMI-A (120 μM and 240 μM, dissolved in 2 μl PBS) were implanted on top of the CAM. CAMs were examined daily until day 12 and photographed *in ovo* with a Zeiss SR stereomicroscope equipped with the MC63 Camera System (Zeiss, Oberkochen, Germany). Blood vessels entering the sponge within the focal plane of the CAM were counted by two observers in a double-blind fashion at 50×. At day 12, CAMs were processed for light microscopy. Briefly, the embryos and their membranes were fixed *in ovo* in Bouin's fluid, dehydrated in graded ethanols, embedded in paraffin, serially sectioned at 7 μm, according to a plane perpendicular to their free surface, stained with a 0.5% toluidine blue, and observed under a light microscope.

The angiogenic response was evaluated as microvessel density in the CAM area under the implant. The area occupied by microvessels was estimated by using a morphometric method of ‘point counting’ (Ribatti *et al*, 1995). Briefly, every third section with 30 serial slides from an individual specimen was analysed simultaneously by two investigators by a 144-intersection point-square reticulum of 0.125 mm^2^ inserted in the eyepiece of the double-head light microscope. Six randomly chosen microscopic fields of each section were evaluated at 250× for the total number of the intersection points that were occupied by vessels transversally cut (diameter ranging from 3 to 10 μm). Mean values±standard deviation (s.d.) for vessel counts were determined for each analysis. The vascular density was indicated by the final mean number of the occupied intersection points, expressed as a percentage of the total number of intersection points.

## RESULTS

### Effect of NAMI-A on endothelial cells

**Figure 1:**
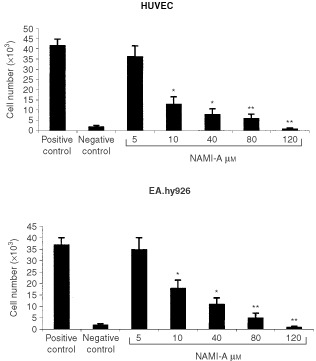
Effect of NAMI-A upon endothelial cell proliferation. Low density cultures of endothelial cells (2.5×10^3^ well^−1^) were exposed on day 0, 2 and 4 with complete medium (positive control), serum-free medium (negative control) or complete medium containing different NAMI-A concentrations. Cell number was evaluated on day 6 by Kueng *et al*'s method (1989). Bars, means±s.d. of three independent experiments. *P<0.05 and **P<0.01, Student–Newman–Keuls analysis of variance.

To evaluate the effects of NAMI-A upon endothelial cell proliferation, HUVEC or EA.hy926 cells were exposed on days 0, 2 and 4 to complete medium (positive control), or to this medium admixed with different NAMI-A concentrations, or to the starvation SFM (negative control), and their proliferation rate was measured on day 6 by a colorimetric method. NAMI-A induced a dose-dependent inhibitory effect on endothelial cell proliferation (Figure 1

). In the concentration range between 5 and 80 μM, endothelial cells were not able to proliferate as controls although maintaining their viability, thus indicating that NAMI-A produced a cytostatic effect. In contrast, the cell number at 120 μM was significantly lower than the number of cells cultured in the SFM, thus indicating that NAMI-A induced cell death. Signs of cytotoxicity, namely vacuolisation, nuclear fragmentation and homogenisation, cellular shrinking or membrane blebbing, loss of elongated shape and cell detachment were indeed detected at 120 μM.

**Figure 2:**
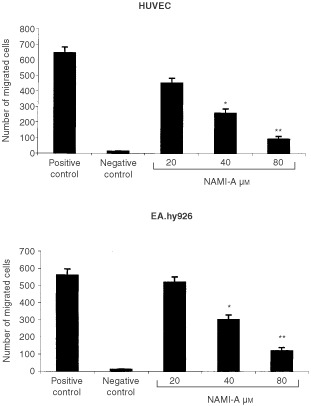
Effect of NAMI-A upon endothelial cell chemotaxis. 1.2×10^5^ cells, exposed for 24 h to each dose of NAMI-A, were seeded in the upper compartment of the Boyden chamber, and the conditioned medium of NIH3T3 cells was placed in the lower compartment as the chemoattractant. Unexposed cells were used in the positive and negative control, respectively, the latter being devoid of the chemoattractant. Cells that migrated after 6 h incubation to the lower surface of the filter separating the compartments were counted. Bars, means±s.d. of the number of migrated cells in five to eight 400x fields of three filters per specimen. *P<0.05 and **P<0.01, Student–Newman–Keuls analysis of variance.

Both HUVEC and EA.hy926 cells, pre-treated for 24 h with different concentrations of NAMI-A and left to migrate towards the NIH3T3 CM chemo-attractant in the Boyden chambers, showed a progressive dose-dependent inhibition of migration which rose 50% of the positive control at 40 μM (Figure 2

).

**Figure 3:**
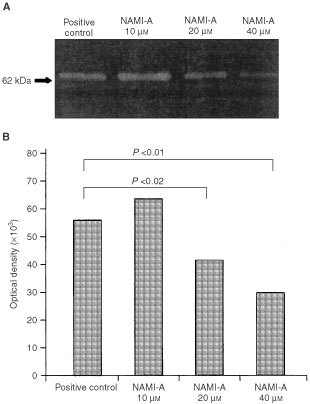
Effect of NAMI-A on MMP-2 production by EA.hy926 cells. MMP-2 activity was evaluated from a densitometric scan of bands resulting on a SDS/polyacrylamide/gelatin gel loaded with the conditioned medium of untreated (positive control) or NAMI-A-treated EA.hy926 cells.

Lastly, EA.hy926 cells were exposed to increasing concentrations of NAMI-A for 18 h, and their CM were tested for the presence and activity of MMP-2 by gelatin zymography and gelatinolysis scoring. Unexposed cells gave MMP-2 gelatinolytic band with an apparent relative molecular mass of 62 kDa (Figure 3A

), indicating that the enzyme was constitutively secreted and readily activated (Van Wart and Birkedal-Hansen, 1990). In this form, MMP-2 persisted in CM even after exposure to NAMI-A, but its secretion declined significantly at 20 μM and further at 40 μM (Figure 3B).

Importantly, inhibition of chemotaxis and MMP-2 production occurred without inhibition of endothelial cell proliferation. Cell number after 18- and 24-h NAMI-A treatment (40, 120 and 240 μM) was not statistically different from controls (data not shown).

### Effect of NAMI-A on angiogenesis *in vivo*

The CAMs (20 per series) were examined macroscopically on the incubation day 12. CAMs implanted with the angiogenic factor FGF-2 (positive control) displayed a vasoproliferative response in form of allantoic vessels spreading radially towards the sponge in a spoked wheel pattern (Table 1

**Table 1:**
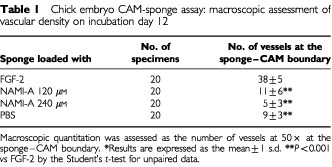
Chick embryo CAM-sponge assay: macroscopic assessment of vascular density on incubation day 12

and Figure 4A

**Figure 4:**
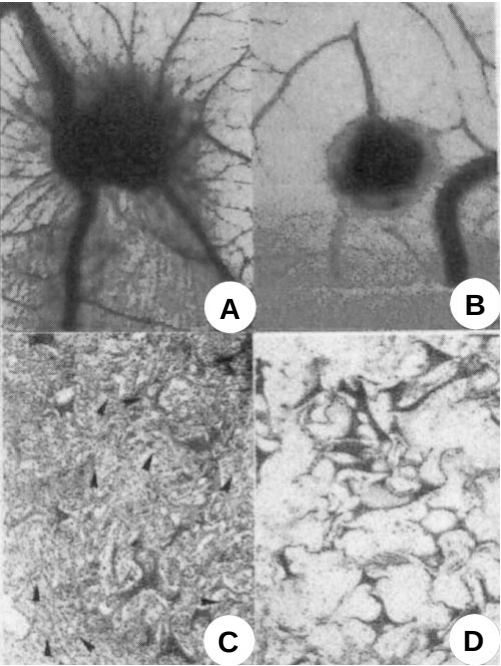
The CAM of a 12-day-old chick embryo incubated for 4 days with a gelatin sponge loaded with (**A**), (**C**) the angiogenic fibroblast growth factor-2 (FGF-2) or with (**B**), (**D**) 240 μM of NAMI-A. Note in (**A**) numerous blood vessels converging like spokes toward the sponge, whereas in (**B**) there are very few vessels around the sponge or converging toward it. (**C**) Histologic section of the sponge shows numerous vessels (arrows) intermingled in a collagenous matrix among the trabeculae. (**D**) No vessels are detectable. Original magnifications: **A**, **B**, 50×; **C**, **D**, 400×.

). When the sponge was loaded with PBS, physiologic angiogenesis was observed as fewer allantoic vessels arranged partly around the sponge and partly converging towards it (Table 1). By contrast, very few vessels were detectable with 120 μM and even fewer with 240 of μM NAMI-A (Table 1 and Figure 4B).

Histologic examination and planimetric vessel counting were also performed (Table 2

**Table 2:**
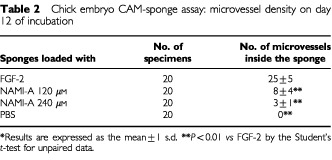
Chick embryo CAM-sponge assay: microvessel density on day 12 of incubation

). FGF-2-loaded sponges displayed a dense collagenous matrix and numerous blood vessels among the sponge trabeculae (Table 2 and Figure 4C). By contrast, very few or no vessels could be detected inside the PBS- and NAMI-A-loaded sponges (Table 2 and Figure 4D).

## DISCUSSION

NAMI-A was repeatedly described to inhibit lung metastasis growth of solid metastasising murine tumours (Sava *et al*, 1998a, 1999b; Zorzet *et al*, 2000) by a mechanism unrelated to tumour cell direct cytotoxicity (Sava *et al*, 1998a; Bergamo *et al*, 1999).

Here we show NAMI-A to inhibit a number of *in vitro* endothelial cell functions, namely proliferation, chemotaxis and matrix metalloproteinase production. These functions are essential steps for neovessel sprouting (Kubota *et al*, 1988; Connolly *et al*, 1989) and angiogenesis, which is an important biological process involved in metastasis formation (Liotta *et al*, 1991; Weidner *et al*, 1991). Importantly, throughout the experiment with the CAM model, this study showed NAMI-A to prevent *in vivo* angiogenesis at doses known to have anti-metastasis activity. In fact, NAMI-A concentration in the lung of mice bearing pulmonary metastases, following a treatment cycle (35 mg kg day^−1^ for six consecutive days) active on metastasis growth, is in the range 100–300 μM (Sava *et al*, 1998b, 1999b; Cocchietto and Sava, 2000).

*In vitro* inhibitory effects of NAMI-A on endothelial cell functions are dose-dependent and are obtained at non-cytotoxic concentrations. In fact, the 50% inhibitory concentration of NAMI-A on both HUVEC and EA.hy926 cell proliferation is about 10 μM, i.e. a concentration more than 10-fold lower than the cytotoxic one. Interestingly, the inhibition of endothelial cell proliferation required a rather long exposure of 144 h. No such effect occurred with shorter challenges (up to 96 h), and on other cell types such as TS/A, MCF7, B16F10 and KB NAMI-A showed a marginal cytostatic effect at mM concentrations whereas 10–100 μM NAMI-A were completely devoid of cell toxicity (Fnd Callerio, data on file). Similarly, the inhibitory effects upon chemotaxis and matrix metalloproteinase production occurred in the absence of any inhibition of cell proliferation or of cytotoxicity, thus indicating the absence of a general toxic effect on the treated cells and suggesting a peculiar mode of action of NAMI-A which deserves further investigation; studies by Pintus *et al*, at the Department of Biochemical Sciences of the University of Sassari, Italy, seem to confirm this hypothesis, showing NAMI-A active on the MAPK ERK1/2 pathway (G Pintus, personal communication to G Sava).

NAMI-A inhibits chemotaxis of endothelial cells and particularly the FGF-2-induced chemotaxis, since the NIH3T3 CM applied as chemoattractant contains FGF-2 as the prominent stimulatory factor (Rusnati and Presta, 1996). It also inhibits the production of MMP-2 by EA.hy926 cells. This effect appears unrelated to a general reduction of protein synthesis, since NAMI-A never did show such effect on the treated cells (Bergamo *et al*, 1999). However, although the evaluation of the treated cells by flow cytometry or by the sulforhodamine B test often showed an increase of cell proteins, the possibility of the selective inhibition of focal protein synthesis can not be excluded. MMP-2 secretion involves the degradation of type IV, V, VII and X collagens and fibronectin (Mullins and Rohrlich, 1983) which are constituents of both the basement membrane and the interstitial stroma (Tryggvason *et al*, 1987). This degradation facilitates the intrusion of endothelial cells across such structures, an important step for neovessel sprouting (Mullins and Rohrlich, 1983; Tryggvason *et al*, 1987) and metastasis formation (Tryggvason *et al*, 1987; Mignatti and Rifkin, 1993). Also, previous results by this group showed the inhibition of mRNAs for MMP-2, specularly to the increase of the mRNA for TIMP-2, to be associated to enhanced deposition of extracellular matrix within the tumour, increased thickness of the capsule surrounding it and appearance of a more defined wall around tumour blood vessels (Sava *et al*, 1996); all these events were matched to a marked reduction of the formation of spontaneous metastases.

NAMI-A can directly inhibit the enzymatic activity of MMP-2 and MMP-9, as shown by the zymography performed with conditioned medium of fibrosarcoma (HT-1080) and neuroblastoma (SK-N-BE) tumour cells (data not shown). From the experimental data, the 50% inhibitory concentration of NAMI-A can be estimated around 1–1.5 mM. This concentration is about 10-times higher than that obtained in tumour masses after a complete cycle of *in vivo* treatment, therefore a direct effect of NAMI-A upon matrix metalloproteinase activity appears irrelevant.

Importantly, the concentrations of NAMI-A active *in vitro* on endothelial cell functions, and effective *in vivo* in the CAM model, are easily reached from the beginning of the schedule of anti-metastasis treatment. In fact, considering that (i) 24 h after the sixth injection, NAMI-A is about 0.2 mM in the lungs, the site of metastasis growth, (ii) tissue distribution from the blood compartment is rather fast immediately after injection (Sava *et al*, 1999b; Cocchietto and Sava, 2000), and that (iii) the half-life time for elimination after repeated treatments is about 20 h (Cocchietto and Sava, 2000), ruthenium concentration in these tissues after the first injection is not less than one half of that found at the end of treatment.

Therefore it may be supposed that the finding that ‘active’ concentrations may persist *in vivo* in the mouse lungs for up to 48 h after last drug injection should warrant the exposure of the tumour cells present in these tissues for a time long enough to induce effects on the metastatic cells even greater than those presently observed *in vitro*.

In conclusion, this study demonstrates that NAMI-A can be credited with anti-angiogenic activity, this property probably contributing to its widely described *in vivo* anti-metastasis effect.
